# Assembly and comparative analysis of the complete mitochondrial genome of *Salix wilsonii* using PacBio HiFi sequencing

**DOI:** 10.3389/fpls.2022.1031769

**Published:** 2022-11-16

**Authors:** Fuchuan Han, Yanshu Qu, Yicun Chen, Li’an Xu, Changwei Bi

**Affiliations:** ^1^ Key Laboratory of Forestry Genetics & Biotechnology of Ministry of Education, Co-Innovation Center for Sustainable Forestry in Southern China, Nanjing Forestry University, Nanjing, China; ^2^ Research Institute of Subtropical Forestry, Chinese Academy of Forestry, Hangzhou, China; ^3^ College of Information Science and Technology, Nanjing Forestry University, Nanjing, China

**Keywords:** *Salix wilsonii*, mitochondrial genome, comparative analysis, HiFi sequencing, assembly

## Abstract

*Salix* L. (willows) is one of the most taxonomically complex genera of flowering plants, including shrubs, tall trees, bushes, and prostrate plants. Despite the high species diversity, only five mitochondrial genomes (mitogenomes) have been released in this genus. *Salix wilsonii* is an important ornamental and economic willow tree in section *Wilsonia* of the genus *Salix*. In this study, the *S. wilsonii* mitogenome was assembled into a typical circular structure with a size of 711,456 bp using PacBio HiFi sequencing. A total of 58 genes were annotated in the *S. wilsonii* mitogenome, including 33 protein-coding genes (PCGs), 22 tRNAs, and 3 rRNAs. In the *S. wilsonii* mitogenome, four genes (*mttB*, *nad3*, *nad4*, and *sdh4*) were found to play important roles in its evolution through selection pressure analysis. Collinearity analysis of six *Salix* mitogenomes revealed high structural variability. To determine the evolutionary position of *S. wilsonii*, we conducted a phylogenetic analysis of the mitogenomes of *S. wilsonii* and 12 other species in the order Malpighiales. Results strongly supported the segregation of *S. wilsonii* and other five *Salix* species with 100% bootstrap support. The comparative analysis of the *S. wilsonii* mitogenome not only sheds light on the functional and structural features of *S. wilsonii* but also provides essential information for genetic studies of the genus *Salix*.

## Introduction

Mitochondria are semiautonomous organelles that originated from symbiotic bacteria within eukaryotic cells (Gupta and Golding, 1996; van Oven and Kayser, 2009). They have established a stable regulatory mechanism with the nuclear genome during long-term evolution. The nucleus plays a dominant role in the cell, while the growth of mitochondria is controlled by the nuclear genome and its genetic system. Plant mitochondria, the main sites of aerobic cellular respiratioFabaceae differ by approximatelyn, not only provide energy for complex intracellular biological activities but also are involved in lipid metabolism, cytoplasmic inheritance, and apoptosis (Green and Reed, 1998; Olson and Stenlid, 2001; McBride et al., 2006; Osiewacz et al., 2010). With the development of sequencing technologies, especially HiFi sequencing that balances length and high accuracy, increasing numbers of plant mitogenomes are being assembled and released. This development also drives progress in plant mitogenome research and provides important data for obtaining deeper insight into the structure, evolution, and function of mitogenomes (Palmer et al., 2000; Guo et al., 2016; Cole et al., 2018).

The mitogenome of plants is more complex in structure than that of other eukaryotes. In addition to a master circular molecule, it also includes linear conformations, branched structures, and numerous small circular molecules mediated by repeat sequences (Notsu et al., 2002; Kozik et al., 2019; Li et al., 2022). For example, the mitogenome of *Populus simonii* was assembled into three circular molecules, and all of them had protein-coding capability (Bi et al., 2022). Additionally, the sweet potato (*Ipomoea batatas* [L.] Lam) mitogenome was found to have a multicircular structure generated by repeat sequence-mediated recombination (Yang et al., 2022). The mitogenome of plants is much larger than that of other eukaryotes and differs even among related species (Kubo and Newton, 2008; Sloan et al., 2012). The largest terrestrial plant mitogenome is 11.3 Mb in size, while the smallest is approximately 66 kb (Sloan et al., 2012; Skippington et al., 2015). Additionally, the sizes of the mitogenomes of *Medicago truncatula* (271 kb) and *Phaseolus vulgaris* (588 kb) in the Fabaceae differ by approximately 300 kb (Bi et al., 2020). Studies have shown that foreign sequence insertions and numerous repeat sequences are the main causes of mitogenome expansion in plants (Richardson and Palmer, 2007; Li, 2009; Smith, 2011). Previous studies of *Acer truncatum*, *Malus domestica*, *Cucumis sativus*, and melon detected a significant number of sequences that are homologous to nuclear and chloroplast sequences (Alverson et al., 2011; Rodríguez-Moreno et al., 2011; Goremykin et al., 2012; Ma et al., 2022). Moreover, a study in *Cucumis sativus* revealed that numerous repeat sequences contribute significantly to the expansion of the mitogenome (Lilly and Havey, 2001; Alverson et al., 2011). In terms of genomic content among land plants, mitochondrial intron counts vary from only 4 in *Viscum scurruloideum* (Skippington et al., 2015) to >40 in hornworts (*Anthoceros agrestis* and *Anthoceros angustus*) (Gerke et al., 2020), while the number of protein-coding genes (PCGs) ranges from as few as 19 in *Viscum scurruloideum* to 41 in the liverworts *Marchantia polymorpha* (Oda et al., 1992) and *Scapania ornithopodioides* (Dong et al., 2019). The differences in gene numbers, intron counts, and intergenic contents of plant mitogenomes are probably caused by intracellular and horizontal transfer processes, which enable the integration of foreign DNA. However, to maintain functional stability, the number of genes in the mitogenome varies very little between species. These features suggest that the instability of plant mitogenomes has led to a significant lag in studies on them compared with the chloroplast and plastid genomes.

*S. wilsonii*, commonly known as Ziliu in China, is a deciduous tree that can grow up to 13 m tall. It belongs to the genus *Salix*, which represents one of the most taxonomically complex genera of flowering plants. It is frequently used as a horticultural plant due to its purple branches, and it is a fast-growing, economically valuable tree whose branches are frequently used for weaving (Kuzovkina et al., 2008; Wu et al., 2019). However, the assembly of plant mitogenomes has proven challenging because of numerous repeat sequences, intricate structure, and insertions of foreign sequences. In this work, PacBio HiFi reads were used to obtain a high-quality assembly of the *S. wilsonii* mitogenome. Comparative analysis of the *S. wilsonii* mitogenome is imperative for better elucidating the functional and structural features of *S. wilsonii*, which would facilitate evolutionary and genetic studies of *Salix*.

## Materials and methods

### Plant material and DNA sequencing

Materials were collected from fresh leaves of *S. wilsonii* on the campus of Nanjing Forestry University (32°04’41” N, 118°48’23” E). The fresh leaves were placed in a -80°C freezer for freezing and storage. DNA was extracted from the leaves. After the quality of the isolated DNA was checked, the libraries were constructed using the SMRTbell Express Template Preparation Kit 2.0 (Pacific Biosciences, CA, USA). A SMRTbell library was obtained after quality control testing. The library was sequenced on the PacBio Sequel II platform (Pacific Biosciences, CA, USA) (PacBio Sequel II System).

### Assembly and annotation of the mitogenome

PacBio SMRT-Analysis software (https://www.pacb.com) was used for quality control of the raw polymerase reads. The obtained CCS reads were used to generate an assembly with Hifiasm v0.16 software (Cheng et al., 2021). All generated contigs were aligned to the reference mitogenomes of *Salix suchowensis* and other *Salix* species using BLASTn (Camacho et al., 2009), and finally a circular contig was found to be the *S. wilsonii* mitogenome sequence. The online tool GE-seq (https://chlorobox.mpimp-golm.mpg.de/geseq.html) (Tillich et al., 2017) was applied to annotate the *S. wilsonii* mitogenome, using the *Salix purpurea* and *S. suchowensis* mitogenomes as reference genomes. The threshold for protein, rRNA, tRNA, and DNA search identity was 85%. Subsequently, the annotation results were edited using Apollo to manually modify the GenBank file (Lewis et al., 2002). The circular map of the *S. wilsonii* mitogenome was visualized using the OrganellarGenomeDRAW program (Greiner et al., 2019).

### Identification of repeat sequences

Simple sequence repeats (SSRs) were identified using MISA v2.1 (Beier et al., 2017) (https://webblast.ipk-gatersleben.de/misa/). We identified units of 1-6 bp with the minimum number of repeats set to 8, 4, 4, 3, 3, and 3, respectively. The online version of Tandem Repeats Finder 4.09 (Benson, 1999) (https://tandem.bu.edu/trf/trf.html) was used to identify tandem sequence repeats based on default parameters. Dispersed repeats were searched by the online version of REPuter with the parameters minimal repeats and hamming Distance set to 30 and 3 bp, respectively (Kurtz et al., 2001) (http://bibiserv.techfak.uni-bielefeld.de/reputer/), and the results were verified by BLASTn (evalue < 1e-10, identity > 80) (Camacho et al., 2009).

### Selection pressure analysis of PCGs

We calculated nonsynonymous (Ka) and synonymous (Ks) substitution rates for 23 PCGs of *S. wilsonii*. *Arabidopsis thaliana* (NC_037304.1), *Manihot esculenta* (NC_045136.1), *Passiflora edulis* (NC_050950.1) and *Populus tremula* (NC_028096.1) were used as reference species in this study. Twenty-three PCGs of the reference species and *S. wilsonii* were compared using ParaAT 2.0 (default parameters) (Zhang et al., 2012). Subsequently, Ka/Ks values were calculated using KaKs_Calculator v.2.0 (Wang et al., 2010).

### Collinearity analysis

To study the collinearity between *S. wilsonii* and other members of the genus *Salix*, high-quality mitogenomes were downloaded from the NCBI (https://www.ncbi.nlm.nih.gov/genome) with the accession numbers shown in **Table 1**, which included *Salix brachista, Salix cardiophylla, Salix paraflabellaris, Salix purpurea*, and *Salix suchowensis* (Huang et al., 2019; Chen et al., 2020). Sequence alignment of *S. wilsonii* and the above species was performed using the subroutine nucmer of Mummer 3 (Marçais et al., 2018). The collinearity results were then filtered using the subroutine delta filter in Mummer 3 (identity>90, length>50). The collinearity results were statistically analyzed and visualized using ggplot2 (Wickham, 2016).

**Table 1:** Genomic features of six *Salix* mitogenomes.

Species Name	Accession number	Genome Size (bp)	Gene number				GC%	AT Skew	GC Skew
			Full	PCGs	rRNA	tRNA			
*S. wilsonii*	NC_064688.1	711,456	58	33	3	22	44.83	0.001	-0.002
*S. brachista*	NC_058733.1	608,983	59	33	3	23	44.93	-0.005	-0.005
*S. cardiophylla*	NC_052708.1	735,173	56	28	3	25	44.80	-0.003	-0.004
*S. paraflabellaris*	NC_046754.1	637,893	55	30	3	22	44.92	0	-0.004
*S. purpurea*	NC_029693.1	598,970	55	32	2	20	44.94	-0.011	-0.001
*S. suchowensis*	NC_029317.1	644,437	58	33	3	22	44.98	-0.003	0.007

### Phylogenetic analysis

To analyze the evolutionary position of *S. wilsonii* in the order Malpighiales, all released mitogenomes of Malpighiales in the NCBI (https://www.ncbi.nlm.nih.gov/genome) were selected for evolutionary analysis in this study. The species whose mitogenomes were selected included members of the Salicaceae (*S. wilsonii*, *S. brachista*, *S. cardiophylla*, *S. paraflabellaris*, *S. purpurea*, *S. suchowensis*, *P. alba*, *P. davidiana*, and *P. tremula*), Rhizophoraceae (*Bruguiera sexangula*; NC_056359.1), Euphorbiaceae (*M. esculenta*) and Passifloraceae (*P. edulis*). Additionally, *A. thaliana* was selected as an outgroup. The common PCGs among the 14 selected species were identified using Python scripts. The protein sequences were then subjected to multiple sequence alignment using MAFFT v7.475 (Katoh and Standley, 2013). Conserved regions of the aligned sequences were extracted using the Gblocks v0.91b default parameter (Castresana, 2000). The maximum likelihood (ML)-based evolutionary tree was constructed using IQ-TREE v2.1.2 with 1000 bootstraps, and the GTR+F+R2 model was selected according to Bayesian information criterion scores (Minh et al., 2020).

## Results

### Structural characteristics of the *S. wilsonii* mitogenome

**Figure 1:**
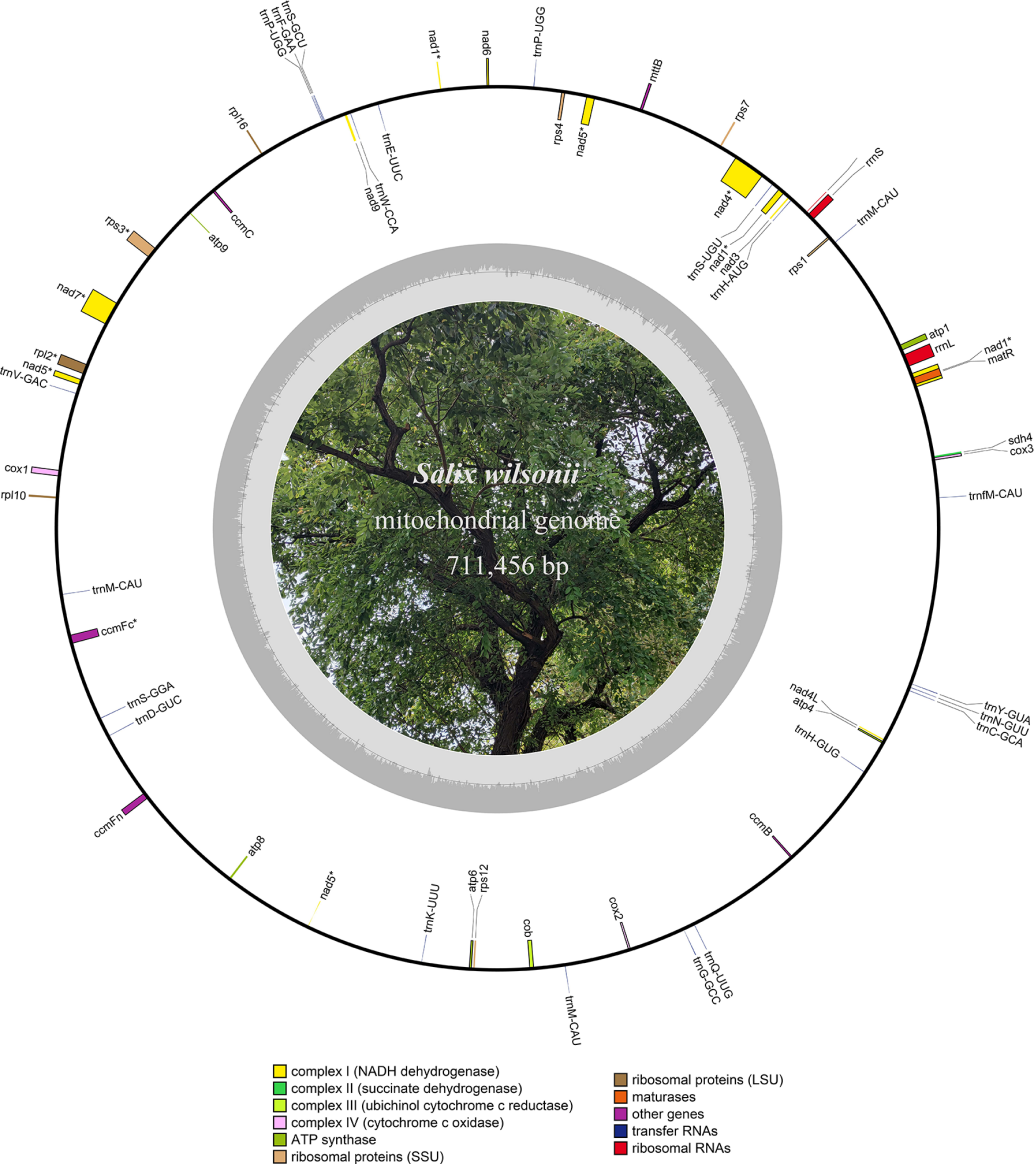
Circular map of the *S. wilsonii* mitogenome. The inner circle in gray represents the GC content of the chromosome.

The HiFi reads were assembled into a typical circular structure 711,456 bp in size and submitted to NCBI under accession NC_064688.1 (**Figure 1**). In this study, the *S. wilsonii* mitogenome was annotated with a total of 58 genes, including 33 PCGs, 22 tRNA genes, and 3 rRNA genes (**Table 1**). The 33 PCGs consisted of 9 NADH dehydrogenase genes (*nad1*, *nad2*, *nad3*, *nad4*, *nad4L*, *nad5*, *nad6*, *nad7*, and *nad9*), 5 ATP synthase genes (*atp1*, *atp4*, *atp6*, *atp8*, and *atp9*), 4 cytochrome C biogenesis genes (*ccmB*, *ccmC*, *ccmFc*, and *ccmFn*), 3 cytochrome C oxidase genes (*cox1*, *cox2*, and *cox3*), a transport membrane protein (sdh4), a succinate dehydrogenase (mttB), a ubiquinol cytochrome c reductase (*cob*), a maturase (*matR*), 3 ribosomal protein (LSU) genes (*rpl2*, *rpl10*, and *rpl16*), and 5 ribosomal protein (SSU) genes (*rps1*, *rps12*, *rps3*, *rps4*, and *rps7*). The total length of the 33 PCGs was 30,298 bp, accounting for 4.26% of the *S. wilsonii* mitogenome; the total lengths of tRNA and rRNA genes accounted for 0.23% and 0.75%, respectively, while the total length of the intergenic region was close to 95% of the total length. Because the *S. wilsonii* mitogenome has no large repeat regions, all annotated PCGs and rRNA genes are single-copy genes, with only two tRNA genes having multiple copies (*trnM-CAU* and *trnP-UGG*). There were eight PCGs containing introns in the *S. wilsonii* mitogenome (*nad1*, *nad2*, *nad4*, *nad5*, *rps3*, *nad7*, *rpl2*, and *ccmFc*) (**Table 2**).

**Table 2:** Gene composition of the *S. wilsonii* mitogenome.

Group of Genes	Gene Name
NADH dehydrogenase	nad1*, nad2*, nad3, nad4*, nad4L, nad5*, nad6, nad7*, nad9
ATP synthase	*atp1, atp4, atp6, atp8, atp9*
Cytochrome c biogenesis	ccmB, ccmC, ccmFc*, ccmFn
Cytochrome c oxidase	*cox1, cox2, cox3*
Maturases	*matR*
Ubiquinol cytochrome c reductase	*cob*
Ribosomal proteins (LSU)	rpl10, rpl16, rpl2*
Ribosomal proteins (SSU)	rps1, rps12, rps3*, rps4, rps7
Transport membrane protein	*mttB*
Succinate Dehydrogenase	*sdh4*
Ribosomal RNAs	*rrn5, rrnL, rrnS*
Transfer RNAs	*trnC-GCA, trnD-GUC, trnE-UUC, trnF-GAA, trnG-GCC, trnH-AUG*,
	*trnH-GUG, trnK-UUU, trnM-CAU* (*×3*)*, trnN-GUU, trnP-UGG* (*×2*)*, trnQ-UUG*,
	*trnS-GCU, trnS-GGA, trnS-UGU, trnV-GAC, trnW-CCA, trnY-GUA, trnfM-CAU*

*Labeled intron containing genes.

The GC content of *S. wilsonii* was 44.98% (A: 27.62%, C: 22.38%, G: 22.46%, and T: 27.55%), similar to that of other species in the genus *Salix* (**Table 1**). The positive AT skew in the mitogenome of *S. wilsonii* and the negative GC skew indicate a higher content of A and C bases. Thirty-one PCGs had ATG as the start codon, and the three stop codons used were TAA (54.55%), TAG (24.24%), and TGA (21.21%) (**Supplementary Table 1**). However, the start codons of *mttB* and *rpl6* remain unclear and need to be verified experimentally.

### Repeat sequence analysis of the *S. wilsonii* mitogenome

**Figure 2:**
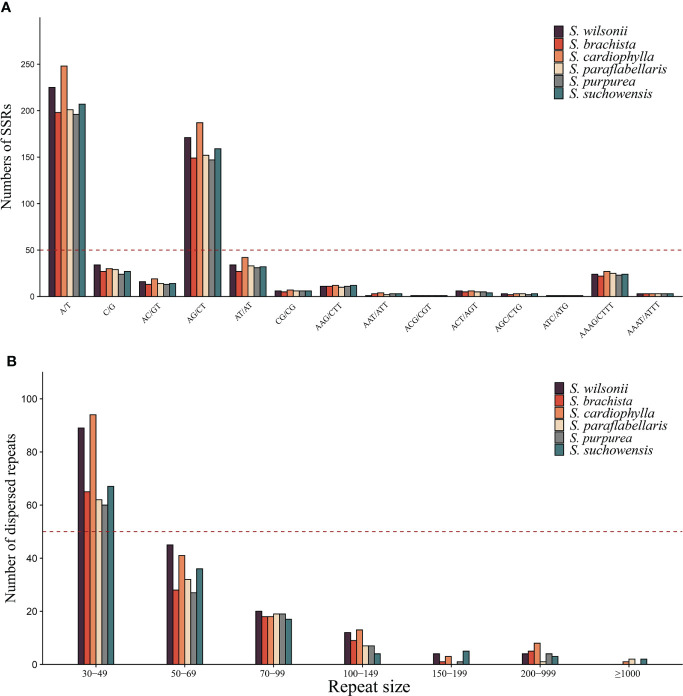
Repeat sequences of the genus Salix. **(A)** Identification frequencies of SSRs with different repeat types in the genus *Salix*. **(B)** The number of dispersed repeats of different lengths in the genus. *Salix*.

SSRs are sequences usually consisting of 1-6 bp unit repeats and are widely distributed in plant mitogenomes (Powell et al., 1996). A total of 608 SSRs were found in the *S. wilsonii* mitogenome, with 259 mononucleotide repeats (42.6%) and 227 dinucleotide repeat (37.34%) being more abundant and 19 pentanucleotide (3.13%) and 4 hexanucleotide repeats (0.66%) being less abundant (**Supplementary Table 2**). The *S. wilsonii* mitogenome had the highest number of A/T repeats (225), accounting for 86.9% of all mononucleotide repeats, which was similar to findings for other *Salix* mitogenomes (Ye et al., 2017). Notably, AG/CT had the highest frequency of the dinucleotide repeats among all *Salix* mitogenomes (**Figure 2A**). Additionally, there was a fairly high frequency of AAAG/CTTT types among the tetranucleotide repeats (**Figure 2A**).

Tandem repeats are unstable in organisms, frequently mutate, are involved in the regulatory activities of the genome, and are closely related to genome recombination and rearrangement (Hannan, 2012). Tandem repeats were found to exist mainly in the intergenic regions of *S. wilsonii*, consisting of 10-30 bp (**Supplementary Table S3**). Most of them were present as two copies, except for the tandem repeat sequence that appears in the *nad1* and *trnS-UGU* interval, with 4.2 copies.

Unlike tandem repeats, dispersed repeat sequences are distributed evenly throughout the mitogenome and promote or repress gene expression in the near-insertion site. Moreover, genes distributed in dispersed repeat sequences are more likely to display multiple copies, such as *trnM-CAU* and *trnP-UGG*. A total of 182 dispersed repeat sequences (21,658 bp) were found in the *S. wilsonii* mitogenome, accounting for 3% of the mitogenome. Eighty-nine dispersed repeat sequences were between 30 and 49 bp in length (48.9%), 45 dispersed repeat sequences were 50-69 bp in size (24.7%), and only two dispersed repeat sequences were >200 bp in size (**Figure 2B**). Dispersed repeat sequences varied considerably between species of the genus *Salix*, with only three species (*S. cardiophylla*, *S. paraflabellaris*, and *S. suchowensis*) having repeat sequences >1,000 bp.

### Selection pressure analyses of mitochondrial PCGs

**Figure 3:**
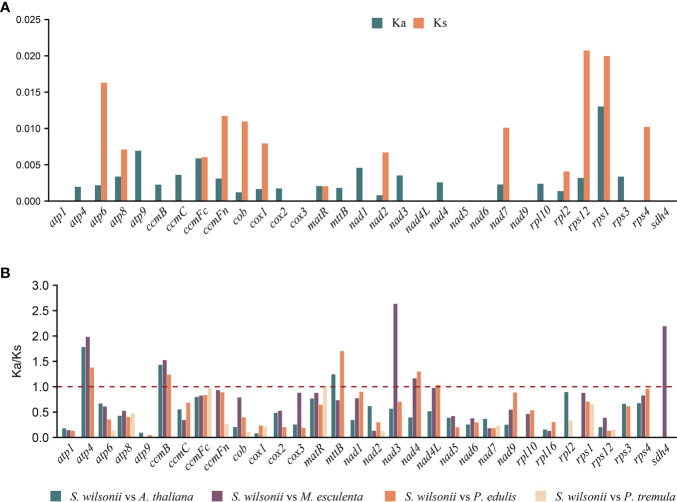
Nonsynonymous (Ka), synonymous (Ks), and Ka/Ks substitution values of *S. wilsonii*. **(A)** Values of Ka and Ks for *P. tremula* vs. *S. wilsonii.* **(B)** Ka/Ks values of 24 PCGs of *S. wilsonii* versus four other species.

The nucleotide substitution rates of mitochondrial PCGs are useful for inferring the direction and magnitude of natural selection acting on homologous PCGs during the evolution of diverged species (Li et al., 1985). A Ka/Ks ratio >1 implies positive or Darwinian selection (driving change), a ratio of exactly 1 indicates neutral selection, and a ratio of <1 implies purifying or stabilizing selection (acting against change). Mitochondria are important sites for cell metabolism, apoptosis, and energy production. To maintain mitochondrial function, deleterious mutations are removed by purifying selection during natural selection; therefore, the Ka value in most genes is smaller than the Ks value (Hurst, 2002; Shtolz and Mishmar, 2019). Twenty-four PCGs from the *S. wilsonii* mitogenome were compared with those from the mitogenomes of 4 other species, namely, *A. thaliana*, *M. esculenta*, *P. edulis*, and *P. tremula* (NC_ 028096.1), and the Ka/Ks ratio was calculated using the YN method in KaKs_Calculator v2.0. Most of the pairwise Ka/Ks ratios were <1 (**Figure 3B**), suggesting that most PCGs were under stabilizing selection during the evolution of *S. wilsonii*. These PCGs with Ka/Ks <1 may play important roles in stabilizing the normal function of mitochondria. In contrast, several genes were also found with Ka/Ks ratios >1 in most species, namely, *atp4*, *ccmB*, *mttB*, *nad3*, *nad4*, and *sdh4*, indicating that they had been under positive selection during evolution. In particular, the *nad3* gene had an extremely high Ka/Ks ratio (*S. wilsonii* vs. *M. esculenta*: 2.63), indicating strong positive selection during the evolution of *S. wilsonii* and *M. esculenta* (**Figure 3B**). Additionally, the Ka and Ks values for most genes of *P. tremula* and *S. wilsonii* were close to 0, indicating a short time of differentiation between them (**Figure 3A**).

### Collinearity analysis of six *Salix* mitogenomes

**Figure 4:**
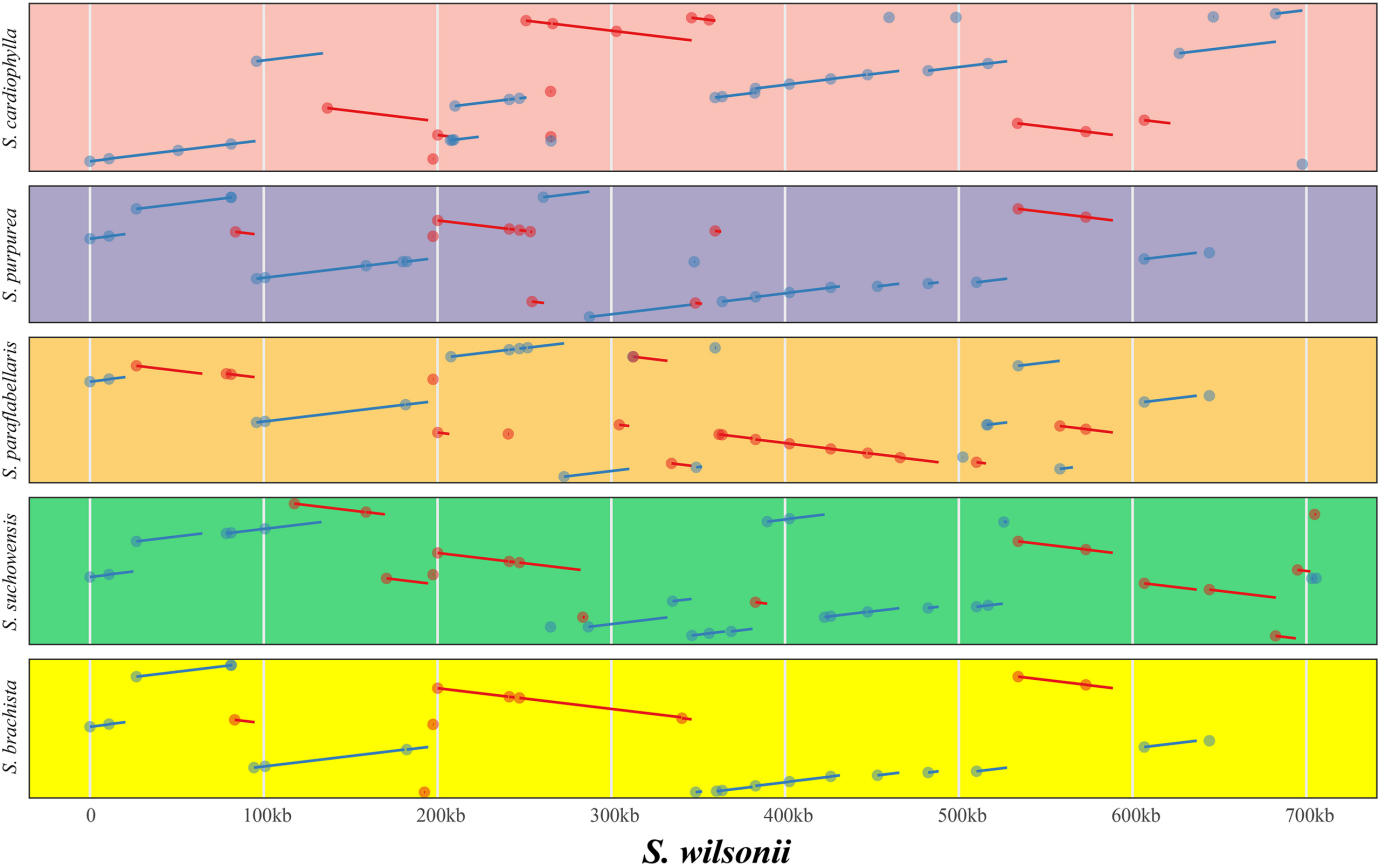
Covariance analysis of six different species in the genus *Salix* using *S. wilsonii* as the reference genome. The pink, purple, gold, green, and yellow represent the covariate regions of *S. wilsonii* vs. *S. cardiophylla*, *S. wilsonii* vs. *S. purpurea*, *S. wilsonii* vs. *S. paraflabellaris*, *S.wilsonii* vs. *S. suchowensis*, and *S. wilsonii* vs. *S. brachista*, respectively. The red and blue lines indicate forward and inverted covariance, respectively.

Genome rearrangement resulting from repeat sequences is a major cause of the evolution of plant mitogenomes. The mitogenome of *S. wilsonii* was compared with those of five other species, namely, *S. brachista*, *S. cardiophylla*, *S. paraflabellaris*, *S. purpurea*, and *S. suchowensis*, using the nucmer program of MUMmer v3.23. **Table 3** shows that there is strong collinearity between *S. brachista* and *S. wilsonii*. The 25 local colinear blocks (LCBs) between them make up 96.48% (587,518 bp) of the *S. brachista* mitogenome, and 99.83% (24,726 bp) of the *S. brachista* PCGs are in LCBs. Additionally, the mitogenomes of *S. paraflabellaris* (LCBs: 25, genome percentage: 92.97%, CDS percentage: 95.47%), *S. purpurea* (LCBs: 16, genome percentage: 90.33%, CDS percentage: 98.06%), and *S. suchowensis* (LCBs: 20, genome percentage: 94.27%, CDS percentage: 98.19%) also showed good collinearity relationships with the *S. wilsonii* mitogenome, with their LCBs accounting for more than 90% of their mitogenomes. Interestingly, there was poor collinearity between *S. cardiophylla* and *S. wilsonii*, but the protein-coding regions showed stronger collinearity, further suggesting that the PCGs are more stable. The dot plot indicates that there is frequent rearrangement in *Salix* mitogenomes (**Figure 4**), which may be related to numerous recombination events occurring in repeated sequences during evolution.

**Table 3:** Collinearity features between *S. wilsonii* and other five *Salix* mitogenomes.

Species	Genome Size (bp)	CDS Length (bp)	Collinearity			
			Length (bp)	Numbers	Genome Percent (%)	CDS Percent (%)
*S. brachista*	608,983	29,556	587,518	25	96.48	99.83
*S. cardiophylla*	735,173	24,726	645,494	26	87.80	96.38
*S. paraflabellaris*	637,893	26,245	593,048	25	92.97	95.47
*S. purpurea*	598,970	29,631	541,024	16	90.33	98.06
*S. suchowensis*	644,437	30,200	607,520	20	94.27	98.19

### Horizontal transfer of sequences from the chloroplast genome

**Figure 5:**
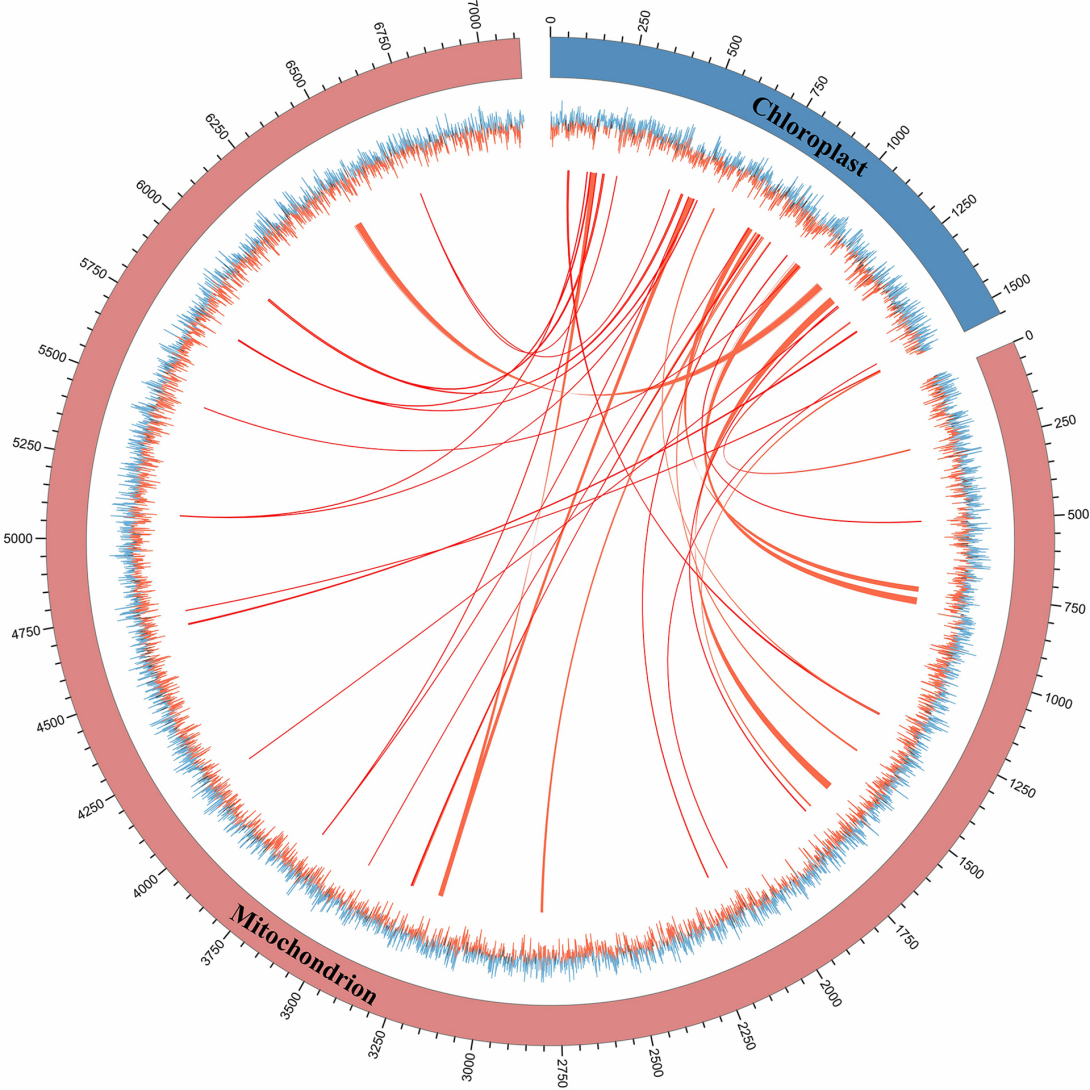
The shared transfer sequence of the mitogenome and chloroplast genome of *S. wilsonii*. The folded line represents the GC skew of the mitogenome and chloroplast genome. The linkage indicates the shared transfer sequence between the mitogenome and the chloroplast genome.

The phenomenon of horizontal sequence transfer occurs frequently between organelles and is an important cause of mitogenome expansion. In this study, multiple chloroplast-derived sequence transfer events in the *S. wilsonii* mitogenome were identified (Wu et al., 2019). **Figure 5** shows numerous shared transfer sequences between the chloroplast genome and the mitogenome of *S. wilsonii*. A total of 43 DNA fragments with a total length of 23,368 bp were derived from the chloroplast genome, accounting for 3.28% of the *S. wilsonii* mitogenome (**Supplementary Table 4**), which was a very common proportion of the known angiosperm mitogenomes (Wang et al., 2019). A total of six sequences were longer than 1,500 bp in size, with the longest fragment measuring 2,800 bp. Among the chloroplast-derived sequences, four complete chloroplast PCGs (*psaA*, *psbC*, *psbD*, and *psbH*) and seven complete tRNA genes (*trnp-UGG*, *trnD-GUC*, *trnM-CAU*, *trnN-GUU*, *trnV-GAC*, *trnV-UAC*, and *trnW-CCA*) were observed. Additionally, our results also demonstrated that some PCGs, i.e., *rrn16*, *psbB*, *clpP*, *psbA*, *rpoC2*, *rps7*, *rps12*, and *accD*, migrated from the chloroplast genome to the mitogenome in *S. wilsonii* (**Supplementary Table 4**), and most of them lost their integrity during evolution.

### Phylogenetic analysis of the mitogenome

**Figure 6:**
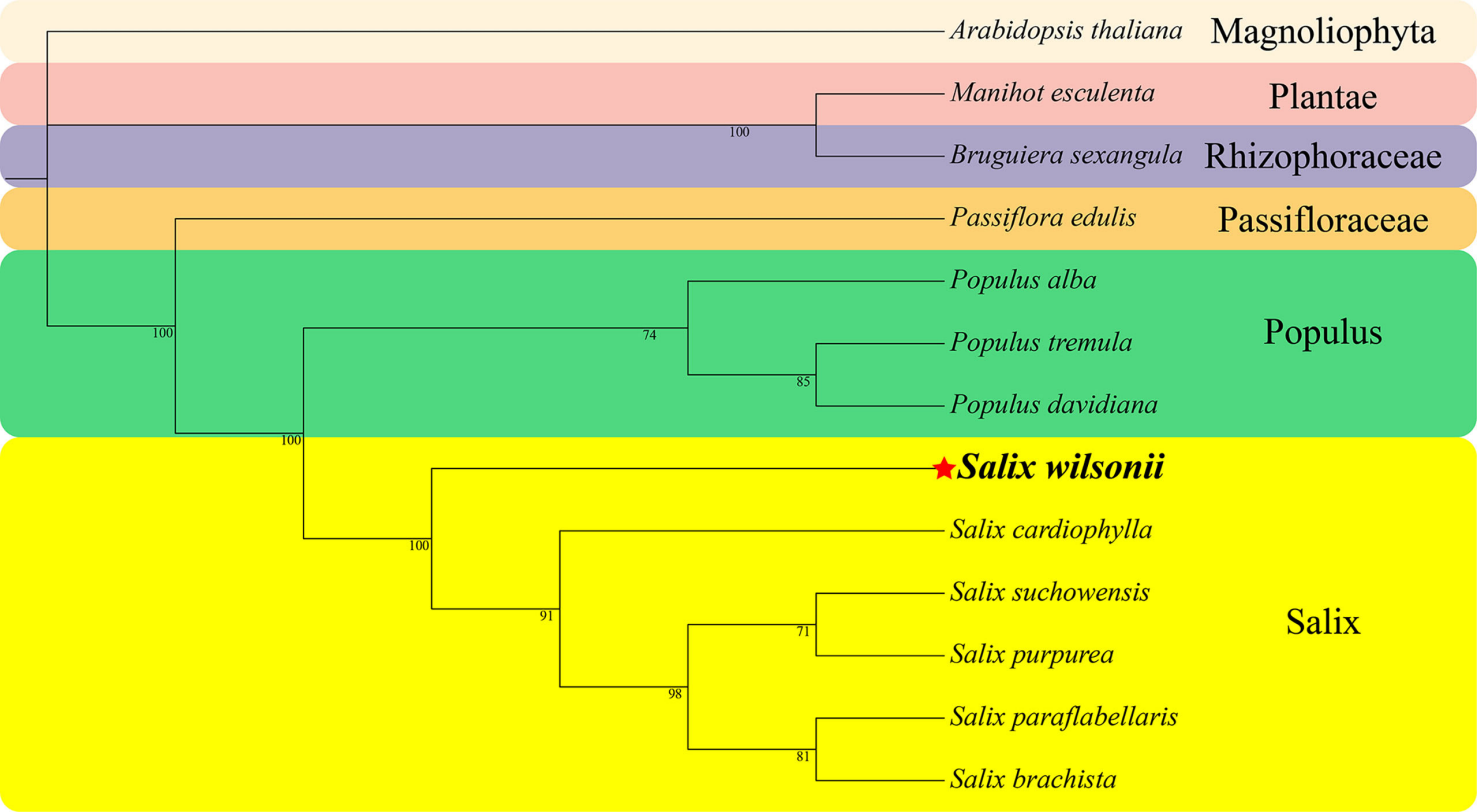
The phylogenetic relationships of Malpighiales. The number on each branch is the bootstrap support value. Colors indicate families of each species, where *Populus* and *Salix* of Salicaceae are indicated.

A phylogenetic analysis based on the conserved PCGs of *S. wilsonii* and 12 other species of Malpighiales, including 8 Salicaceae (5 *Salix* and 3 *Populus*), one Passifloraceae, one Rhizophoraceae, one Plantae, and one Magnoliophyta species, was performed (**Figure 6**). The phylogenetic tree strongly supported the segregation of *S. wilsonii* and five other *Salix* plants with 100% bootstrap support, as well as the segregation of *Salix* and *Populus* (100%) and the segregation of Passifloraceae and Salicaceae (100%). In addition, the Passifloraceae clustered with the Euphorbiaceae, which is consistent with traditional phylogenetic relationships (Group et al., 2016).

## Discussion

Due to the limitations of DNA sequencing technology, only 14 plant mitogenomes were released before 2005, and most of them were assembled by Sanger capillary sequencers (Jansen et al., 2005), such as those of *Marchantia polymorpha* (Oda et al., 1992), *Nicotiana tabacum* (Sugiyama et al., 2005), and some algae. Benefiting from the emergence of next-generation sequence (NGS) and TGS technology, as well as some brilliant assembly tools, an increasing number of mitogenomes of cash crops have been released, including those of *Oryza sativa*, *Zea mays*, *Sorghum bicolor*, and *Cucumis sativus* (Clifton et al., 2004; Tian et al., 2006; Alverson et al., 2011). However, limited by the short sequencing length of NGS technology and the high error rate of TGS technology, the *de novo* assembly of complex plant mitogenomes is challenging. With the rise of the PacBio HiFi sequencing method, which yields highly accurate long-read sequence datasets, it has rapidly become the ‘gold’ standard for the *de novo* assembly of genomes. Most published *Salix* mitogenomes were assembled by the next-generation sequencing data, which may result in incorrect assembly of some regions (**Supplementary Table 5**). In this research, the first *de novo* assembly of the *S. wilsonii* mitogenome was completed using PacBio HiFi sequence technology, providing a reference for its genetic study.

The plant mitogenome is a dynamically changing entity during evolution, showing great variation in structure and size among species (Bi et al., 2022). For this reason, studies of the plant mitogenome lag far behind those of the chloroplast genome. As of Aug. 2022, only 453 plant mitogenomes have been released in the NCBI Organelle Genome Database (https://www.ncbi.nlm.nih.gov/genome/), but over 7,400 chloroplast genomes have been released. Plant mitogenomes contain a large number of repeat sequences and foreign sequence insertions, resulting in gene loss and multiple copies, while repeat sequences mediate genomic rearrangements that also form multichromosomal structures. Studies have shown that 71.5% of the *Malus domestica* mtDNA sequence is highly similar to its nuclear DNA sequence and is the driving force of its mitogenome expansion (Goremykin et al., 2012). The melon (*Cucumis melo* L.) mitogenome is over 2.7 Mb in size, eight times larger than that of other cucurbits, and contains a large number of repeat sequences and a high content of nucleus-derived DNA, accounting for 42% and 47% of the total sequence (Rodríguez-Moreno et al., 2011), respectively. The high frequency of recombination mediated by three pairs of long repeats in the okra (*Abelmoschus esculentus*) mitogenome results in four molecules existing at the same time (Li et al., 2022). *Acer truncatum* and *Glycine max* contain numerous foreign sequences, and most genes with transferred sequences are tRNA genes (Chang et al., 2013; Ma et al., 2022).

**Figure 7:**
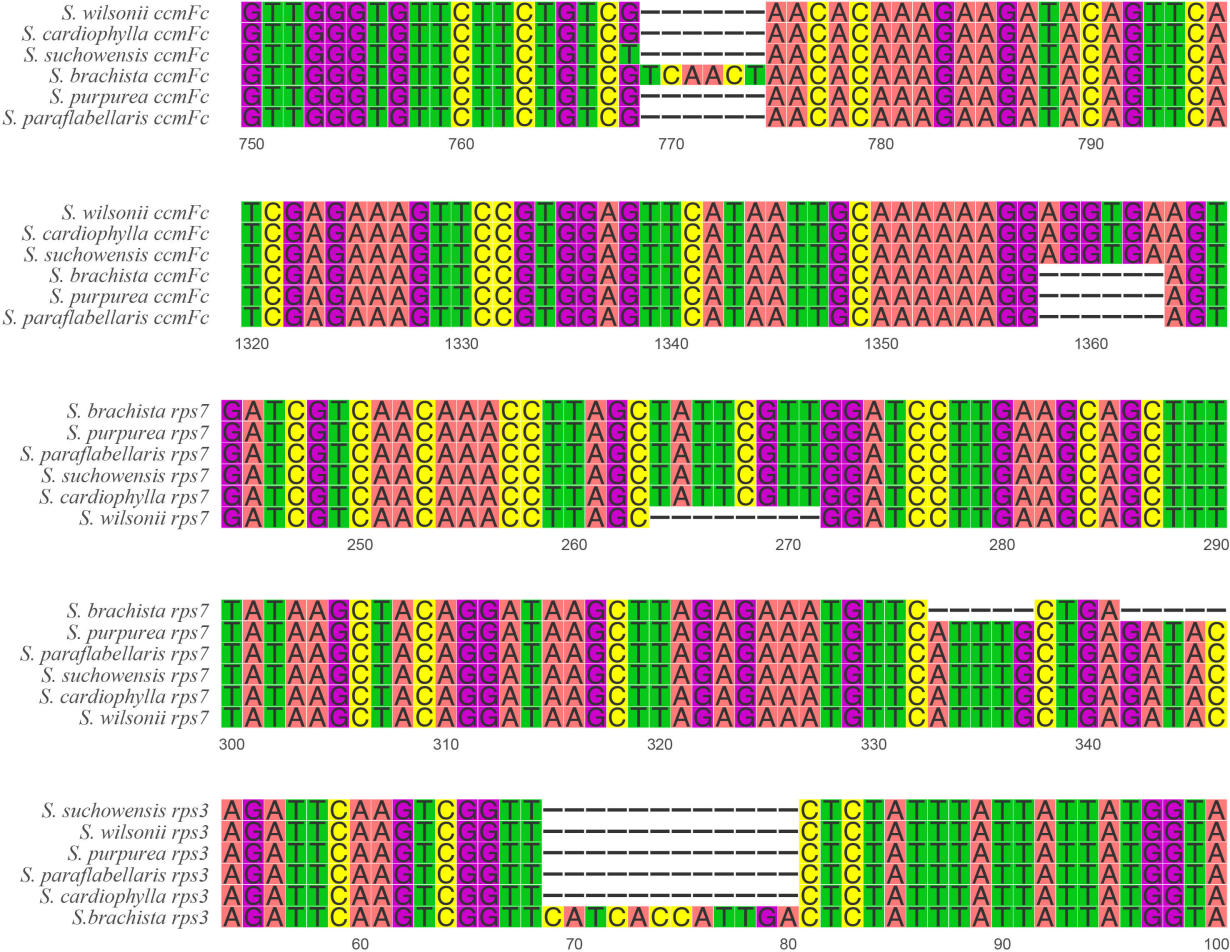
InDel distribution of PCGs in six *Salix* mitogenomes.

The size of the mitogenome varies considerably in the genus *Salix* (**Table 1**), ranging from 735 kb (*S. cardiophylla*) to 599 kb (*S. purpurea*). Comparative analysis of the repeat sequences of the six *Salix* mitogenomes revealed that the mitogenomes of *S. cardiophylla* and *S. wilsonii* were larger and had more repeat sequences. This result further indicates the importance of repeat sequences for the expansion of plant mitogenomes. The numbers of genes and PCGs in the mitogenome were not positively correlate. In the genus *Salix*, the number of genes ranged from 55 to 59, and the number of PCGs ranged from 28 to 33 (**Table 1**). However, *S. cardiophylla* had the fewest PCGs, which contrasts with it having the largest mitogenome. Repeat sequence rearrangements can also result in gene loss and multiple copies (Bi et al., 2016; Liao et al., 2018; Cheng et al., 2021). However, the PCGs in the *S. wilsonii* mitogenome are all single-copy genes, and only the tRNA genes have multiple copies. There are no repeat sequences >300 bp in size in the *S. wilsonii* mitogenome, which may contribute to the stability of the genome structure and gene contents. In addition, five InDels were found in three mitochondrial PCGs (*ccmFc*, *rps3*, and *rps7*) in the genus *Salix* (**Supplementary Table 6**). The *rps7* of *S. wilsonii* and *S. brachista* had 8 and 5 bp deletions forming a shift mutation, respectively (**Figure 7**), which may result in the loss of gene functions. In addition to repeat sequences, insertions of foreign sequences can also significantly affect the expansion of the mitogenome. Studies have shown that tRNA genes lost from mitochondria during evolution are generally compensated for by transferred sequences from chloroplasts (Woodson and Chory, 2008). Similar to the results for the *A. truncatum* mitogenome, the sequences in the *S. wilsonii* mitogenome transferred from the chloroplast genome were mostly tRNA genes. In conclusion, repeat and foreign sequences influence the expansion of the mitogenome and even affect some important functions.

Repeated sequences mediate genomic rearrangements, causing significant variation in mitogenomes among species. Studies have proven that mitogenome structure is highly diverse in the genus *Populus*, with the mitogenomes of *Populus simonii* and *Populus deltoides* showing a multicircular structure (Bi et al., 2022; Qu et al., 2022) but those of *Populus alba* and *Populus davidiana* both having a single circular chromosome. The current findings indicate that all *Salix* mitogenomes have a single circular chromosome, but the collinearity within the genus *Salix* is not as strong as that with the genus *Populus* (Bi et al., 2022), suggesting rich species diversity in *Salix*. Notably, there was strong collinearity in the protein-coding regions within the genus *Salix*, a result similar to that in *C. vulgaris* (Turmel et al., 2003). The results further prove that the structure of the plant mitogenome is complicated and variable but that the sequences of its PCGs are highly conserved.

During evolution, most PCGs in mitochondria are relatively conserved, contributing to the maintenance of normal mitochondrial function. Analysis of selection pressure showed that *atp4*, *ccmB*, *mttB*, *nad3*, *nad4*, and *sdh4* were subject to positive selection (Ka/Ks > 1) after ancestral differentiation (**Figure 3**). Additionally, the *sdh4* gene is only present in *S. wilsonii* and *M. esculenta* among the five compared species, indicating that *sdh4* is evolutionarily unstable and was frequently lost from mitogenome during evolution (Adams et al., 2001; Petersen et al., 2017).

## Conclusions

The application of PacBio HiFi sequencing technology, which combines long reads and high accuracy, has rapidly changed previous sequencing strategies. In this study, the complete mitogenome of *S. wilsonii* was assembled and comparatively analyzed using PacBio HiFi sequencing. By comparative genomic analysis of the mitogenome of *S. wilsonii*, we determined the phylogenetic relationships of *S. wilsonii*. In addition, we inferred that a large number of repeat sequences and foreign sequences from the chloroplast genome are the main reasons for the expansion of the *S. wilsonii* mitogenome. The more conserved PCG region was further demonstrated by collinearity analysis, showing its contribution to the functional stability of mitochondria. In conclusion, this study of the mitogenome of *S. wilsonii* provides important information for evolutionary studies of *S. wilsonii*.

## Data availability statement

The original contributions presented in the study are publicly available. This data can be found here: NCBI, PRJNA880582.

## Author contributions

FH: bioinformatic analyses, writing of original draft, writing of review, and editing. YQ: data curation, resources, and software. LX: validation and funding acquisition. YC: validation and formal analysis. CB: conceptualization, funding acquisition, supervision, writing of review and editing. All authors contributed to the article and approved the submitted version.

## Funding

The work is supported by the Natural Science Foundation of Jiangsu Province (BK20220414) and the Natural Science Foundation of the Higher Education Institutions of Jiangsu Province. The work is also supported by the National Key Research and Development Program of China (2021YFD2201205) and the Priority Academic Program Development of Jiangsu Higher Education Institutions (PAPD).

## Acknowledgments

The authors sincerely thank laboratory members for assistance with the study.

## Conflict of interest

The authors declare that they have no known competing financial interests or personal relationships that could have appeared to influence the work reported in this paper.

## Publisher’s note

All claims expressed in this article are solely those of the authors and do not necessarily represent those of their affiliated organizations, or those of the publisher, the editors and the reviewers. Any product that may be evaluated in this article, or claim that may be made by its manufacturer, is not guaranteed or endorsed by the publisher.
